# The incidence of cardiovascular events after hospitalization due to CAP and their association with different inflammatory markers

**DOI:** 10.1186/1471-2466-14-197

**Published:** 2014-12-12

**Authors:** Olga Rajas, Mara Ortega-Gómez, José María Galván Román, José Curbelo, Guillermo Fernández Jiménez, Lorena Vega Piris, Francisco Rodríguez Salvanes, Belén Arnalich, Sergio Luquero Bueno, Ana Díaz López, Hortensia de la Fuente, Carmen Suárez, Julio Ancochea, Javier Aspa

**Affiliations:** Servicio de Neumología, Hospital Universitario de la Princesa, Instituto de Investigación Sanitaria Hospital Universitario de la Princesa, IP, Madrid, España; Biobanco Instituto de Investigación Sanitaria Hospital Universitario de la Princesa, IP, Madrid, España; Servicio de Medicina Interna, Hospital Universitario de la Princesa, Instituto de Investigación Sanitaria Hospital Universitario de la Princesa, IP, Madrid, España; Unidad de información Clínica, Hospital Universitario de la Princesa, Instituto de Investigación Sanitaria Hospital Universitario de la Princesa, IP, Madrid, España; Unidad Metodológica, Instituto de Investigación Sanitaria Hospital Universitario de la Princesa, IP, Madrid, España; Servicio de Neumología, Hospital Universitario del Henares, Madrid, España; Servicio de Análisis Clínicos, Hospital Universitario de la Princesa, Instituto de Investigación Sanitaria Hospital, Universitario de la Princesa, IP, Madrid, España; Servicio de Inmunología, Hospital Universitario de La Princesa, Madrid, España

**Keywords:** Community-acquired pneumonia (CAP), Cardiovascular events, Inflammatory markers, CAP late mortality

## Abstract

**Background:**

Late prognosis of Community-Acquired Pneumonia (CAP) patients is related to cardiovascular events. Persistence of inflammation-related markers, defined by high circulatory levels of interleukin 6 and 10 (IL-6/IL-10), is associated with a higher post-event mortality rate for CAP patients. However, association between these markers and other components of the immune response, and the risk of cardiovascular events, has not been adequately explored. The main objectives of this study are: 1) to quantify the incidence of cardiovascular disease, in the year post-dating their hospital admittance due to CAP and, 2) to describe the distribution patterns of a wide spectrum of inflammatory markers upon admittance to and release from hospital, and to determine their relationship with the incidence of cardiovascular disease.

**Methods/design:**

A cohort prospective study. All patients diagnosed and hospitalized with CAP will be candidates for inclusion. The study will take place in the Universitary Hospital La Princesa, Spain, during two years. Two samples of blood will be taken from each patient: the first upon admittance and the second one prior to release, in order to analyse various immune agents. The main determinants are: pro-adrenomedullin, copeptin, IL-1, IL-6, TNF-α, IL-17, IFN-γ, IL-10 and TGF-β, E-Selectin, ICAM-1, VCAM-1 and subpopulations of peripheral T lymphocytes (T regulator, Th1 and Th17), together with other clinical and analytical variables. Follow up will start at admittance and finish a year after discharge, registering incidence of death and cardiovascular events. The main objective is to establish the predictive power of different inflammatory markers in the prognosis of CAP, in the short and long term, and their relationship with cardiovascular disease.

**Discussion:**

The level of some inflammatory markers (IL-6/IL-10) has been proposed as a means to differentiate the degree of severity of CAP, but their association with cardiovascular risk is not well established. In this study we aim to define new inflammatory markers associated with cardiovascular disease that could be helpful for the prognosis of CAP patients, by describing the distribution of a wide spectrum of inflammatory mediators and analyzing their association with the incidence of cardiovascular disease and mortality one year after release from hospital.

## Background

Community acquired pneumonia (CAP) is an important worldwide health problem. It is the most frequent infectious disease requiring hospitalization. The Incidence of CAP in Europe ranges from 1.2 to 11.6 cases per 1000 of population per year, with rates of hospitalization ranging from 40 to 60% [1, 2].

The mortality rate of CAP during hospitalization is well known. But various authors have reported an increased mortality rate after a long-term follow-up of hospitalized, Community-Acquired Pneumonia (CAP) patients who survived the illness. It has even been observed after allowing for age and co-morbidity. This tendency has been related to the Pneumonia Severity Index (PSI) risk scale [3–9].

It is known that significant cardiac complications are produced in a large proportion of CAP patients [10–13], with a greater incidence at the outset of the event, but also during its evolution and follow-up. Perry et al. [14], in connection with a very broad sample (50,119 subjects), recently published that a significant number of patients, with follow-up during 90 days after admittance for CAP, experienced a cardiovascular event, principally during hospitalization, with a rate of 1.5% for myocardial infarction, 10.2% for congestive heart failure, 9.5% for arrhythmia, 0.8% for unstable angina and 0.2% for ictus. Other authors previously had already described this relationship [15, 16].

Likewise Jasti et al. [17] described how the majority of readmissions after CAP resulted from cardio-pulmonary or underlying neurological diseases. Corrales-Medina et al. [18] also described the association between acute bacterial pneumonia and acute coronary syndrome. Besides, the relationship between coagulation disorders and CAP has also been reported [19].

Epidemiological data have shown a strong relationship between acute respiratory infections and a later myocardial infarction [20, 21]. Furthermore, it is known that chronic inflammation destabilizes atheroma plaque and induces a pro-coagulant state [22]. Very recently it has been described that an important number of patients have new cardiac arrhythmia during and post-hospitalization for pneumonia [23].

Altogether these observations suggest that the way of regarding CAP should be changed: early diagnosis, better monitoring, and risk stratification in CAP with respect to cardiovascular diseases should be established.

A growing interest has developed in identifying different biological markers suitable for establishing a correct diagnosis and adequately evaluating the severity and prognosis of the disease [24–27]. Various candidate biomarkers have been studied, including inflammatory molecules such as C-reactive protein, IL-6, IL-1 and procalcitonin and also cardiovascular biomarkers such as proatrial natriuretic peptide (pro-ANP), proarginin-vasopresin (copeptin), proendothelin-1 and Mid-regional pro-adrenomedullin (MR-Pro-ADM). These cardiovascular biomarkers are good predictors, both in the short and in the long term, for the prognosis of CAP [26]. In a reference study, MR-pro-ADM resulted to be the most useful biomarker [25]. Moreover, it also was an excellent marker to predict short and long term mortality in CAP, regardless of its etiology [28].

It has been reported that persistent high circulatory levels of IL-6 and IL-10 after CAP are associated with an increased mortality in the follow-up [29, 30], pointing to a relationship between a persistent inflammation upon release from hospital and the long-term prognosis. Moreover, in apparently healthy subjects, high levels of IL-6 are related with a greater risk of myocardial infarction in the future [31]. It remains to be established whether these inflammatory mediators could be used as biomarkers for prognosis of cardiovascular risk in CAP patients.

Immune response is very complex and many other molecules might be used as biomarkers. Of great relevance in the long-term inflammatory response against infectious agents is the generation of the T lymphocyte effectors type Th1 and Th17, which are capable of mediating the inflammatory response by means of their cytokine effectors IFN-γ and IL-17 in response to such infectious agents [32, 33]. Likewise, the unregulated action of these cellular populations is involved in numerous autoimmune inflammatory processes [34, 35]. The inflammatory state is negatively regulated by factors such as anti-inflammatory cytokines like IL-10 (already studied in CAP) and TGF-β [36] and by T regulator lymphocytes (Treg). The Treg cell populations, characterized by the expression of certain markers such as the transcription factor FoxP3, are capable of attenuating the inflammatory response mediated by T cells in numerous pathological processes [37]. The balance between all these pro and anti-inflammatory factors determines the inflammatory state of the organism. During inflammatory processes the activation of the vascular endothelium increases the expression of inducible leukocyte adhesion molecules such as ICAM-1, VCAM-1 and E-selectin which mediate the adhesion and extravasation of leukocytes to the inflammation site [38]. During the inflammatory process which affects the endothelial barrier, an increase in the soluble forms of these molecules liberated to the extracellular medium is produced in such a way that their circulatory levels may be indicators of inflammatory processes related with cardiovascular risk [39]. All these mediators of immune response could be considered as candidate biomarkers to evaluate cardiovascular risk and prognosis in CAP.

The current research project intends to quantify the incidence and mortality of cardiovascular disease in adult patients, in the year after hospital admission due to CAP.

Moreover, this project tries to describe a wide spectrum of immune response mediators upon admission to and release from hospital in CAP patients. Many of these mediators have been already studied in CAP but some others will be analyzed for the first time.

Our aim is to establish a profile of the inflammatory state of these patients and determine its possible relationship with the incidence of cardiovascular disease.

## Methods/design

A cohort prospective study to determine the prognosis and the incidence of cardiovascular events after admission to hospital due to a CAP episode and the predictive value of a wide panel of biological inflammatory markers. All the patients will be followed up on for at least one year after discharge and every new cardiovascular event will be recorded. The study will take place in the University Hospital “La Princesa” (HUP), a reference center for emergencies and admissions for three hundred thousand inhabitants in the Northeastern area of Madrid (Spain).

Candidates for this study will be all those patients who are attended to and are subsequently admitted to the hospital due to CAP between 01/01/2014 and 31/12/2015. They must satisfy the following conditions: be older than 18, show symptoms of lower airway infection, together with the existence of fresh radiological infiltrate, and be then diagnosed with pneumonia and subsequently admitted. There must be no alternative diagnoses during follow-up, and the subjects must not have been hospitalized in at least the ten days preceding the current episode. Signed informed consent will be required. The exclusion criteria will concern those patients who are unreachable during the follow-up time.

The Clinical Research Ethics Coimmittee of the hospital has approved this study.

### Main outcomes or primary outcome

The dependent variable in this study is the development of cardiovascular disease according to the following clinical criteria: pacemaker emplacement, acute myocardial infarction, heart failure, acute pulmonary edema, unstable angina, ictus, transitory ischemic cerebral affectation, pulmonary thrombo-embolism and deep venous thrombosis. Moreover, mortality from cardiovascular diseases or any other cause will be registered. These events will be collated during the episode of CAP and during follow-up over one year. Furthermore, the follow-up point at which a cardiovascular event diagnosis or death occurs will be collated with the aim of estimating the incidence in terms of incident density.

### Independent variables

Upon admittance to the emergency department: age, gender, previous antibiotic treatment (type, days, and dosage), vaccination condition (flu and pneumococcal) will be registered as independent variables. In addition, current treatment, especially in relation with cardiovascular disease - antiaggregants, oral anticoagulants, beta-blockers, calcium-antagonists, ACE inhibitors, Angiotensin II receptor blockers, Direct renin inhibitor, mineralocorticoid receptor antagonist, anti-arrhythmics, insulin, oral hypoglucemiants, fibrates, niacin, omega three, fit sterols – will also be recorded.

#### Co-morbidities

Smoking status (packs per year), alcohol consumption, Charlson index [40], peripheral arterial disease (symptomatic or asymptomatic), previous stroke (established or transient ischemic attack), diabetes, obesity, high blood pressure, dyslipemia, chronic kidney disease (glomerular filtration rate –GFR- < 60 ml/m and/or increased high fractional excretion of albumin), liver disease, HIV infection, neoplasic disease, collagen disease, ischemic cardiopathy (MI or revascularization), previous pulmonary thromboembolism or deep venous thrombosis, ICU admission, prior pneumonia and cardiovascular risk evaluation in accordance with the score [41] will all be taken into consideration.

#### During hospitalization

CURB-65 [42], PSI [43], sepsis, septic shock, pleural effusion, empyema, multilobar impairment, hospital stay, admittance to ICU, need for assisted ventilation, microbiological results.

#### Analytical parameters

**Figure 1:**
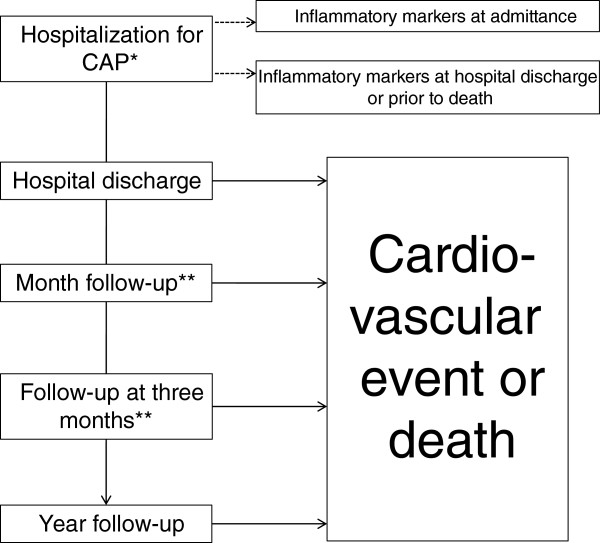
**Flow chart of the study.** *CAP: community adquired pneumonia. **Hospital visit or telephone contact.

Peripheral blood samples will be taken on admittance and near to discharge. The time of the second sample corresponding to release has been standardized to being between the third and the fifth day after the admittance of the patient in order to avoid sample acquisition losses for the project (see Figure 1). This decision was taken based on the effects of the treatment administered to the patient and its repercussions on the inflammatory markers.

In both samples the following will be determinated: cholesterol levels (LDL, HDL), triglycerides, glucose, HbA1c, creatinine, urea, GFR, ions, blood cell counts, c reactive protein, erythrocyte sedimentation, fibrinogen and coagulation. Besides these, several biological markers will be analyzed (see below).

#### Biological markers

One part of the blood sample will be processed to obtain serum which will be frozen at - 80°C in order to study soluble mediator levels. Another part of the blood sample will be used to analyze, by flow cytometry, the sub-populations of the T, Th1, Th17 and T regulator lymphocytes.

1. Soluble Mediator Study: pro-adrenomedullin and copeptin. pro-inflammatory cytokines: IL-1, IL-6, TNF-α, IL-17, IFN-γ. Anti-inflammatory cytokines: IL-10 and TGF-β. Soluble endothelial adhesion molecules: E-Selectin, ICAM-1 and VCAM-1.
  Determination of the levels of these soluble molecules will be carried out using the commercially available kit *BD CBA Cytometric Bead Array (CBA) Human Soluble Protein Flex Set.* This allows the simultaneous quantification, by flow cytometry, of multiple proteins at a very low concentration (10–2500 pg/mL) in serum samples. Flow cytometry will be carried out with a FACs (*Fluorescence-activated cell sorting*) CANTO cytometer and FCAP Array™ Software will be used for the analysis and quantifications. Due to the technical limitations of the trial, the quantification of soluble serum factors will be carried out in batches of 90 stored serum samples.
2. Sub-populations of T Lymphocytes: by using flow cytometry on peripheral blood lymphocytes to detect CD4, the intracellular transcription factor FoxP3, intracellular cytokine IFN-γ and intracellular cytokine IL-17 will be determined. The proportion of T regulatory lymphocytes will be defined as the percentage of lymphocytes CD4 + FoxP3+. The proportion of type Th1 T-lymphocytes in peripheral blood will be determined by the proportion of lymphocytes CD4+ IFN-γ+. The proportion of Th17 T-lymphocytes will be defined by the proportion of lymphocytes CD4 + IL-17 + .
  For the intracellular biomarkers IL-17 and FoxP3 the use of a human Th17/Treg phenotyping kit (BD Human Th17-Treg Phenotyping Kit) has been chosen with the addition of an Anti-Human IFN-γ. Before marking, stimulation of PBMC (*Peripheral Blood Mononuclear Cell*) by means of PMA (*Phorbol 12-myristate 13-acetate*), Ionomycin and GolgiStop™, fixation and permeabilization with specific buffers for FoxP3 and marking using a cocktail of FoxP3, IL-17, IFN-γ and CD4 antibodies will be performed. Its interpretation will be carried out through a FACs CALIBUR cytometer and its analysis by means of the CellQuest Pro program.

### Follow-up after discharge

There will be at least three visits during follow-up: at one month, at three months and at one year (see Figure 1). If the patient does not attend, a follow-up will be carried out by telephone to determine the cause of absence and a questionnaire will be completed after consultation with the relevant primary care doctor. If the patient is admitted to the center, suffers a cardiovascular event or dies, the pertinent information will be recorded according to primary outcome.

### Data collection method

An electronic, data-collection log, integrated into the hospital’s clinical background system, has been specifically developed for the purposes of this study. The demographic and attendance record information in the study’s data base will be obtained from the general information system to avoid duplication. Access to the data collection log will be restricted, by means of user ID and password, to the study’s researchers, and with its back-up copy policy being the same as that for the general system.

## Statistical analysis

### Sample size

Assuming a cumulative cardiovascular event incidence rate of 20% for the first year after hospital release, in order to carry out such an estimate with a confidence interval (CI) of 95% and with an accuracy of 5%, 243 patients would be necessary. Nevertheless, since antecedents for other similar studies could not be found, and, taking into account that relationships with molecules not previously studied are going to be tested, it has been decided that a sample as large as the recruitment time period permits (2 years) will be used, thus also allowing the possible seasonal effect to be controlled. An approximate average of 600 patients per year are attended to in the Emergency Department with a CAP diagnosis, of which 400 per year are admitted to the hospital, according to the records of the centre. Under these circumstances, and allowing for exclusions, it is estimated that it will be possible to comply with the stated objectives within the set timescale. Furthermore, in order to avoid unnecessary work, 2 intermediate sequential analyses will be carried out when at least 50% of the estimated sample have completed their first year of follow-up so that the estimated value for each molecule may be known before the end of the study. An annual cut-off point will be established for the evaluation of results, potential adverse effects and possible publications.

### Statistical analysis

Descriptive analysis will be carried out by quantitative variables which will be described by their measures of central tendency (mean) and dispersion (standard deviation); qualitative variables will be described by their proportion and standard error. Univariate and multivariate logistic regression will be used to analyze the association between the independent variables and cumulative incidence. Also odds ratios, standard errors and the corresponding test hypothesis will be estimated. The analysis of the association between the qualitative variables will be carried out using the chi-square test or Fisher's exact test and comparing the means of different quantitative variables between subgroups will be performed using *t*-test or ANOVA, or their nonparametric equivalent (Mann-Whiney or Kruskal-Wallis).

The dependent variables will also be analyzed with respect to time: firstly by estimating their incidence density and risk functions according to the Kaplan-Meier method with an appropriate hypothesis test (log-rank, Breslow, Tarone or others), and secondly by using Cox regression models after checking proportionality risk hypotheses. In all cases model construction will be done according to the principle of parsimony in order to include a limited number of variables. All values of p < 0.05 will be considered to be statistically significant. In selection processes p <0.10 or p < 0.15 will be used as a threshold value. Statistical analysis will be carried out using the program Stata v12.0 in the Methodology Unit of the *Princesa* Research Institute.

## Discussion

The importance of CAP as a serious health problem, its high cost in terms of morbidity and mortality and its special relationship with the elderly, are widely-recognized clinical facts. Patients hospitalized with a CAP diagnosis display, after admittance, a higher mortality than patients of their same age and degree of co-morbidity, and also show a higher incidence of cardiovascular disease, both in the long and the short term. Thus, new CAP prognosis scores, which take the appearance of cardiovascular events into account, have been proposed [44, 45]. Various studies dealing with the handling of CAP with the help of several biomarkers have also been published [46–48]. Likewise, articles that try to differentiate between the degree of severity of CAP (severe or non-severe) according to the measurable activity of inflammatory markers [49–51], or help to improve patient stratification in accordance with their levels of inflammatory cytokines, have been published [29]. The inflammatory response to infection produced in patients with CAP could be the cause of cardiovascular complications modifying the prognosis of the disease [10]. Therefore, studying inflammation markers could help to monitor cardiovascular risk and prognosis in CAP patients.

Pneumonia is an exuberant sequestration of peripheral neutrophils in the lungs, which is tightly regulated by cascades of cytokines produced by the immune system in response to an invading pathogen [52, 53]. In an ideal scenario, the acute lung inflammation is protective and self-limiting, and once the infection has been controlled, cytokines also function in restoring homeostasis, including the modulation of neutrophil apoptosis. On the other hand, a cytokine storm results in a deleterious inflammation and poor clinical outcome [53]. The contribution of cytokines to systemic inflammatory response, together with plaque instability, sympathetic activation, a pro-coagulatory state and endothelial dysfunction, could lead to myocardial damage, arrhythmia and heart failure [10].

In the current research project we aim to study the distribution, in these patients, of a wide spectrum of immune response mediators upon admittance to and release from hospital and establish their possible relationship with the incidence of cardiovascular disease, and mortality. This will permit the establishment of a profile of the inflammatory state of these patients and determine its association with cardiovascular risk.

Although the exact role of each immune mediator in the inflammatory response during pneumonia is still a subject of ongoing research, it is clear that different inflammatory patterns are elicited by microorganisms [54].

In the present study we plan to analyze a panel of immune/inflammatory mediators that include: pro-inflammatory cytokines (IL-1, IL-6, TNF-α, IL-17, IFN-γ), anti-inflammatory cytokines (IL-10 and TGF-β); soluble endothelial adhesion molecules (E-Selectin, ICAM-1 and VCAM-1) and Th1, Th17 and Treg lymphocytes. Although they are grouped in this way, the classification of cytokines as pro- and anti-inflammatory is not absolute, since many cytokines are capable of exerting both effects depending on a variety of factors [53, 55, 56], and some patterns of cytokine response could best be described based on their concentrations as high, medium and low, rather than their pro-or anti-inflammatory activities [57]. In addition to cytokines, we will study parameters of immune response such as soluble endothelial adhesion molecules, Th1 and Th17 effector lymphocytes and T regulatory lymphocytes not tested previously in CAP. This set of mediators covers a wide range of processes regulating immune response and inflammation. Their analysis will help to characterize an accurate inflammatory profile of CAP patients and identify candidate inflammatory markers of cardiovascular risk in CAP.

The time during follow-up at which samples for the determination of inflammatory mediators should be taken is an important point for this work. At which time point would determinations better predict cardiovascular risk and evolution/outcome? The average lifespan of the markers needs to be considered. Many cytokines involved in this process have a short half-life and they could be reduced shortly after the induction of the inflammatory response to almost undetectable levels. Accordingly, mean cytokine concentrations are generally highest on admission and they decline rapidly over the first few days. Therefore, measurements of blood samples taken at the time of admission might allow us to better assess the risk for a CAP episode. However, whereas a persistent, up-regulated, pro-inflammatory response is associated with deaths due to cardiovascular disease, renal failure, infection, and cancer, persistent low-grade inflammation and immune suppression may play an important role in coronary events, cerebral-vascular events, and repeat bouts of CAP [31, 53, 58–61]. Therefore, when measured at the end of hospitalization, rather than on admission cytokine levels might actually be more predictive of adverse outcomes after hospitalization. In the current study, blood samples for the determination of inflammatory markers will be taken at both time points. In this way we will be able to establish the best time point for each marker.

In this study we aim at describing the distribution of a wide spectrum of inflammatory markers upon the admittance to and release from hospital of CAP patients, and analyze their association with the incidence of cardiovascular disease and mortality one year after release from hospital. In this way new inflammatory markers associated with cardiovascular disease in these patients may be defined. These new markers could be helpful for prognosis. The development of predictive models provides crucial evidence for the transference of laboratory findings to human beings and from clinical research to clinical practice. The possibility of precisely personalizing healthcare interventions seems to be related with the amount of combined factors in forecasting models [62].

## Abbreviations

CAP: Community-acquired pneumonia
CI: Confidence interval
GFR: Glomerular filtration rate
ICAM: Intercellular adhesion molecule
IFN: Interferon
IL: Interleukin
MR-Pro-ADM: Mid-regional pro-adrenomedullin
PSI: Pneumonia severity index
TNF: Tumor necrosis factor
TFG: Transforming growth factor
VCAM: Vascular cell adhesion molecule.
